# Pre- and post-COVID 19 outbreak relationship between physical activity and depressive symptoms in Spanish adults with major depressive disorder: a secondary analysis of the RADAR-MDD cohort study

**DOI:** 10.3389/fpsyg.2024.1436611

**Published:** 2024-11-12

**Authors:** Delia Ibáñez, Elena Condominas, Josep Maria Haro, Iago Giné Vázquez, RADAR-MDD-Spain, Raquel Bailón, Esther Garcia, Spyridon Kontaxis, Maria Teresa Peñarrubia-Maria, Belen Arranz, Raúl Llaosa-Scholten, Lluisa Gardeñes, Matthew Hotopf, Faith Matcham, Femke Lamers, Brenda W. J. H. Penninx, Peter Annas, Amos Folarin, Vaibhav Narayan, Rodrigo Antunes Lima, Sara Siddi, the RADAR CNS consortium

**Affiliations:** ^1^Department of Psychiatry and Psychology, Hospital Clinic of Barcelona, Barcelona, Spain; ^2^Faculty of Medicine, Universitat Autònoma de Barcelona, Barcelona, Spain; ^3^Centro de Investigación Biomédica en Red de Salud Mental (CIBERSAM), Instituto de Salud Carlos III, Madrid, Spain; ^4^Universitat Politécnica de Catalunya, Barcelona, Spain; ^5^Research, Innovation and Teaching Unit, Parc Sanitari Sant Joan de Déu, Sant Boi de Llobregat, Spain; ^6^Aragón Institute of Engineering Research (I3A), Instituto de Investigación Sanitaria de Aragón (IIS Aragón), University of Zaragoza, Zaragoza, Spain; ^7^Centros de Investigación Biomédica en Red en el Área de Bioingeniería, Biomateriales y Nanomedicina (CIBER-BBN), Madrid, Spain; ^8^Microelectrónica y Sistemas Electrónicos, Universidad Autónoma de Barcelona, Bellaterra, Spain; ^9^Consortium for Biomedical Research in Epidemiology and Public Health (CIBERESP), Instituto de Salud Carlos III, Madrid, Spain; ^10^Unitat de Suport a la Recerca Regió Metropolitana Sud, Fundació Institut Universitari per a la Recerca a l’Atenció Primària de Salut Jordi Gol i Gurina (IDIAPJGol), Barcelona, Spain; ^11^Consorcio de Investigación Biomédica en Red de Epidemiología y Salud Pública (CIBERESP), Instituto de Salud Carlos III, Madrid, Spain; ^12^FEA Psicología Clínica, HU Institut Pere Mata, Vendrell, Spain; ^13^Institute of Psychiatry, Psychology and Neuroscience, King’s College London, London, United Kingdom; ^14^South London and Maudsley NHS Foundation Trust, London, United Kingdom; ^15^School of Psychology, University of Sussex, East Sussex, United Kingdom; ^16^Department of Psychiatry, Amsterdam UMC Location Vrije Universiteit Amsterdam, Amsterdam, Netherlands; ^17^Amsterdam Public Health, Mental Health Program, Amsterdam, Netherlands; ^18^H. Lundbeck A/S, Valby, Denmark; ^19^Department of Psychiatry, University of Oxford, Warneford Hospital, Oxford, United Kingdom

**Keywords:** COVID-19, major depressive disorder, depression, depressive symptoms, physical activity

## Abstract

**Aim:**

To evaluate the longitudinal association of sedentary behavior, light and moderate-to-vigorous physical activity (MVPA) participation with depressive symptoms and whether their possible association changed depending on the pandemic phase.

**Methods:**

This longitudinal study conducted secondary analysis from the Spanish cohort of the Remote Assessment of Disease and Relapse – Major Depressive Disorder (RADAR-MDD) study. Depressive symptoms were assessed by the Patient Health Questionnaire (PHQ-8). Sedentary behavior and physical activity were estimated via wrist-worn devices. Linear mixed models evaluated the longitudinal associations of sedentary behavior and physical activity (light and moderate-to-vigorous intensities) with depressive symptoms.

**Results:**

In total, 95 participants (67.5% women, 53.0 [±10.5] years of age on average) were monitored pre-COVID-19 and included in the analyses. Pre-COVID-19, 73.7% of participants presented depression, and, on average, participated in 13.2 (±1.08) hours/day of sedentary behavior, 2.42 (±0.90) hours/day of light physical activity and 23.6 (±19.80) minutes/day of MVPA. Considering all the observations (from November 2019 to October 2020), an additional hour/day of sedentary behavior was longitudinally associated with higher depressive symptoms [*β*std = 0.06, 95% confidence interval (CI) 0.10 to 0.47], whereas an additional hour/day in light physical activity was associated with lower depressive symptoms (*β*std = −0.06, 95% CI −0.59 to −0.15). Time in MVPA was not associated with depressive symptomatology. The association of sedentary behavior and light physical activity with depressive symptoms was significant only during pre-COVID-19 and COVID-19 relaxation periods, whereas during the strictest periods of the pandemic with regards to the restrictions (lockdown and de-escalation), the association was not observed.

**Conclusion:**

Sedentary behavior and light physical activity were longitudinally associated with depressive symptoms in participants with a history of MDD. The incorporation of light physical activity should be stimulated in adults with a history of MDD. Neither sedentary behavior nor light physical activity were associated with depressive symptoms during the most restrictive COVID-19 phases, whereas sedentary behavior (positively) and light physical activity (negatively) were associated with depressive symptoms in persons with MDD before and after the COVID-19 pandemic.

## 1 Introduction

Depressive disorders are the second leading cause of years lived with disability (GBD 2019 Mental Disorders Collaborators, 2022). A severe form of depression is Major Depressive Disorder (MDD). An MDD episode is diagnosed when low mood and anhedonia occur combined with a range of other symptoms, such as changes in sleep quality, appetite, cognitive function, physical inactivity, asthenia, suicide ideation, and feelings of guilt or worthlessness (American Psychiatric Association, 2013; Beratis et al., 2005; Wagner et al., 2020). MDD has a worldwide prevalence of 2470.5 cases per 100,000 individuals; since the COVID-19 pandemic, its prevalence has increased by 27.6% (COVID-19 Mental Disorders Collaborators, 2021). Approximately, 55% of adults who suffer from MDD will develop chronic depression, characterized by periods of recovery and relapse (Hardeveld et al., 2013; Verduijn et al., 2017).

More recently, growing evidence indicates that physical activity can be a valuable protective factor against depression and a treatment that can mitigate depressive symptoms in persons with MDD (Dishman et al., 2021; Hanssen et al., 2018; Minghetti et al., 2018; Nasstasia et al., 2019; Schuch and Stubbs, 2019; Stubbs et al., 2018), whereas time in sedentary behavior has been associated with a higher risk of developing MDD (Hallgren et al., 2018). Nevertheless, a series of limitations restrict the strength and extension of the evidence.

The number of longitudinal studies, especially with multiple monitoring periods, is scarce (Gianfredi et al., 2021; Hallgren et al., 2018, 2020; Keating et al., 2018). Furthermore, the majority of the current evidence is based on questionnaire-based (indirect) assessment of physical activity and sedentary behavior when evaluating their relationship with depression in persons with MDD (Brondino et al., 2017; de Souza Moura et al., 2015; Krogh et al., 2017; Schuch et al., 2016a; Schuch et al., 2016b). The use of direct assessment, such as accelerometer for the estimation of physical activity level and sedentary behavior, result in more precise and detailed information. Particularly, accelerometers measure the time dedicated to different physical activity intensities (e.g., light and moderate-to-vigorous) during a particular week besides not relying on the memory of the person, avoiding recall bias. Finally, it is also unclear whether the COVID-19 pandemic affected the relationships of sedentary behavior and physical activity with depression in adults with a history of MDD.

Therefore, we evaluated the longitudinal association of sedentary behavior and physical activity with depression in patients with a history of recurrent MDD from the Spanish sample of the Remote Assessment of Disease and Relapse - Major Depressive Disorder (RADAR-MDD) cohort study (Matcham et al., 2019, 2022). Moreover, we evaluated whether the longitudinal association of sedentary behavior and physical activity with depression changed depending on the period of the pandemic (pre-COVID-19, COVID-19 lockdown, de-escalation and relaxation).

The RADAR-MDD study provides a unique opportunity to address the aforementioned objectives (Leightley et al., 2021; Siddi et al., 2022; Sun et al., 2020). First, RADAR-MDD followed participants with MDD since before the COVID-19 outbreak including different phases of the pandemic with multiple monitoring periods (lockdown, de-escalation and relaxation). Second, the study contains direct assessment of physical activity and sedentary behavior, which means higher data quality compared to most of the previous investigations (Currier et al., 2020; Hallgren et al., 2018, 2020; Kaseva et al., 2019; Nasstasia et al., 2019; Stevens et al., 2021). Third, RADAR-MDD monitored participants with MDD in Spain which imposed more restrictive measures than other countries related to outdoors activities and more stringent COVID-19 policies have been associated with poorer mental health (Aknin et al., 2022; Hale et al., 2021; Lavalle et al., 2023). These measures were quantified using the stringency index, with Spain, the Netherlands, and the UK registering indices of 85.2, 78.7, and 79.6, respectively, from February to the first of October 2020, as measured by the COVID-19 government response tracker (Mathieu et al., 2020). Therefore, it is possible to evaluate the effects of this level of restrictions on the relationship of sedentary behavior, physical activity with depression.

## 2 Materials and methods

### 2.1 Study design

This is a secondary analysis from the Spanish cohort of the longitudinal RADAR-MDD research project. In summary, RADAR-MDD study is a collaborative research project carried out in Netherlands, United Kingdom, and Spain (Ranjan et al., 2019). This project is part of the Remote Assessment of Disease and Relapse – Central Nervous System (RADAR-CNS) consortium,^1^ aimed at providing real-time multidimensional indicators of symptoms changes and relapse of individuals with three different health conditions: MDD, multiple sclerosis and epilepsy in order to improve treatment and prevention (Matcham et al., 2019).

The RADAR-MDD dataset allows for detailed monitoring of behavioral and depressive symptoms changes in a sample with a history of MDD (Matcham et al., 2022; Ranjan et al., 2019). RADAR-CNS developed the open-source RADAR-Base platform for data collection and storage from wearables and mobile technologies^2^ (Ranjan et al., 2019). Radar-base provides both passive and active data collection using active and passive remote measurement technology (Ranjan et al., 2019).

The project was co-designed and conducted in partnership with service users in the Patient Advisory Board of the RADAR-CNS study (Matcham et al., 2022; Simblett et al., 2020). They were involved in the choice of measures, the timing and issues of engagement (Simblett et al., 2019, 2020). The full protocol for RADAR-MDD has been reported previously (Matcham et al., 2022).

### 2.2 Study population

The current study included participants from the RADAR-MDD study in Spain who were monitored between November 2017 and October 2020 when the COVID-19 wave started (Real Decreto 926/2020, de 25 de octubre, n°286, 2020). Adults diagnosed of recurrent MDD during their life, who were living in Spain were enrolled from a clinical sample of individuals seeking help for a mental health condition (Matcham et al., 2019).

To be eligible for participation in RADAR-MDD, individuals must: (1) have met DSM-5 (Diagnostic and Statistical Manual of Mental Disorders, 5th edition) diagnostic criteria for non-psychotic MDD within the past 2 years; (2) have recurrent MDD (a lifetime history of at least 2 episodes of depression); (3) be willing and be able to complete self-reported assessments via smartphone; (4) be able to give informed consent for participation; (5) Fluent in Spanish or Catalan; (6) existing ownership of Android smartphone or willingness to use an Android smartphone as their only smartphone; (7) aged 18 or older (Matcham et al., 2022).

The exclusion criteria included: (1) have a lifetime history of bipolar disorder, schizophrenia, MDD with psychotic features, or schizoaffective disorders; (2) dementia; (3) history of moderate to severe drug or alcohol dependence within the last 6 months prior to enrolment; (4) history of major medical disease which might impact upon the patient’s ability to participate in normal daily activities for more than 2 weeks (e.g., due to likely hospitalizations or other periods of indisposition); (5) Pregnancy (although once enrolled, becoming pregnant did not result in withdrawal) (Matcham et al., 2022).

No limitations have been applied regarding any treatment they may be receiving, although medication use and other psychological interventions were monitored throughout the course of follow-up. Additionally, participants were eligible to participate regardless of whether they were currently experiencing depression symptoms or not (Matcham et al., 2022).

In Spain, eligible participants were identified through primary and secondary mental health services or through advertisements for the study placed on mental health charity websites, circulars or Twitter notices (Matcham et al., 2022). Informed consent from all potential participants who fulfilled inclusion and exclusion criteria was obtained. The research was conducted in accordance with the Helsinki Declaration of 1975, as revised in 2008.

In total, 155 Spanish participants met the eligibility criteria and were eligible to participate in the RADAR-MDD study (Matcham et al., 2019). All participants provided written consent previous to the enrolment session (Matcham et al., 2019). For enrolment, depression diagnosis was assessed using the Lifetime Depression Assessment – Self-Report (LIDAS) in addition to the review of medical records (Bot et al., 2017; Matcham et al., 2022).

For the current study, the monitoring period was subdivided into four categories as follows (Lavalle et al., 2023; Cataluña. Departament d’ Interior, 2020; Cataluña. Departament de Salut, 2020; España. Jefatura del Estado, 2020; España. Ministerio de la Presidencia, Relaciones con las Cortes y Memoria Democrática, 2020; España. Ministerio de Sanidad, 2020a,b,c):

- Pre-COVID-19 phase: immediately before the first restrictive measure in relation to the COVID-19 pandemic; November 1st, 2019 to March 10th, 2020 (total of 130 days).
- COVID-19 lockdown refers to the period of the national lockdown in Spain from March 11th, 2020 to April 26th, 2020 (total of 46 days). Specifically, on March 11th, 2020, the Government of Catalonia introduced social distancing to fight the spread of COVID-19 (Resolució SLT/704/2020, d’11 de març, n° 8082A, 2020). On March 14, 2020, the Spanish Government established strict lockdown measures after the declaration of the State of Alarm (Real Decreto 463/2020, de 14 de marzo, BOE nª 3,692, 2020).
- COVID-19 de-escalation refers to the period between April 27th, 2020 and June 18th, 2020 (total of 52 days) when restrictions were lifted gradually through four phases implemented by the Spanish government and supplementary measures of the local Catalan government. See the phases below:
  - During phase 0 non-essential businesses were opened by appointment, and citizens were allowed to do outdoor physical activity by time slot based on age (Orden SND/386/2020, de 3 de mayo, BOE n° 123, 2020).
  - Phase 1 meetings with a maximum of 10 people were allowed; only outdoor spaces of bars and restaurants opened, as well as some spaces of culture, museums, and gyms; transfers to a second residence were permitted (Orden SND/399/2020, de 9 de mayo, n° 130, 2020).
  - Phases 2 and 3 time slots were finished and bars and restaurants’ openings were extended even to the indoor areas, with limited capacity; shopping centers opened, public transport restarted working at 100% and the percentage of capacity in cinemas, theatres, museums increased (Orden SND/414/2020, de 16 de mayo, n° 136, 2020).
- COVID-19 relaxation phase: period between June 19th, 2020 and October 16th, 2020 (total of 119 days) when the mots of the restrictions related to COVID-19 pandemic were eased (Resolució INT/1433/2020, 18 de junio, n° 8,160, 2020; Real Decreto 926/2020, de 25 de octubre, n° 286, 2020).

### 2.3 Assessments

#### 2.3.1 Depressive symptoms

Depressive symptoms were assessed using The Patient Health Questionnaire (PHQ-8) every 2 weeks via the project app (active remote measurement technology) (Matcham et al., 2019). The PHQ-8 is an 8-item self-report questionnaire which measures the frequency of depressive symptoms over the preceding 2-week period (Gómez-Gómez et al., 2023; Kroenke et al., 2009). Each item is rated from 0 = “not at all” to 3 = “nearly every day,” producing a range of total scores from 0 to 24 (increasing severity), which was used for the main analyses (Gómez-Gómez et al., 2023; Kroenke et al., 2009).

For descriptive purposes, participants with a score of ≥10 were classified with depression since it is the most recommended cut-off point for “clinically relevant” depressive symptoms in the previous two weeks (Kroenke et al., 2009).

#### 2.3.2 Physical activity and sedentary behavior

Physical activity and sedentary behavior were estimated through Fitbit devices (Fitbit Charge 2 and 3; Fitbit Inc., San Francisco, CA, USA), which were given to participants (Leightley et al., 2021). Participants were asked to wear the Fitbit devices on the wrist of the non-dominant hand for the duration of the monitoring period. Fitbit 2 or 3 wristband devices are a 3-axis accelerometer-based activity trackers (Matcham et al., 2019). This accelerometer model was chosen by the RADAR-CNS project members together with the volunteers for being commercially available, minimally invasive and easy to use (Polhemus et al., 2020; Simblett et al., 2020).

Fitbit uses a proprietary algorithm which converts raw acceleration data into activity counts in 60-s sampling intervals that define activity intensities classifying each minute as being in sedentary, light, moderate, or vigorous activity (Fitbit Inc, 2018; Hartman et al., 2018; Mikkelsen et al., 2020). We used the daily time spent in sedentary, light and moderate-to-vigorous physical activity (MVPA; moderate + vigorous time) intensities. We only included participants with at least 3 days of physical activity registration and a minimum of 8 valid hours per day in each of the monitoring periods (pre-COVID-19, COVID-19 lockdown, COVID-19 de-escalation and COVID-19 relaxation). For each intensity, we calculated the average time per day dedicated to sedentary behaviour and physical activity considering a day with 16 h (24 h – 8 h of sleep).

Among the 155 participants in the study, 95 fulfilled the required monitoring parameters in the pre-Covid phase, 47 during the lockdown, 46 in the de-escalation phase, and 50 in the COVID-19 relaxation phase.

#### 2.3.3 Confounders

Sociodemographic data (age, sex, marital status [with a partner, single, separated/divorced and widowed]) and comorbidity with other medical illness were collected through a questionnaire at baseline.

#### 2.3.4 Assessments completed by participants per study period

Participants were often monitored (i.e., asked to complete questionnaires and had their physical activity tracked via Fitbit, etc.) multiple times during each of the COVID-19 phases (Pre-COVID-19, COVID-19 lockdown, COVID-19 de-escalation, and COVID-19 relaxation). Table 1 presents the average number of times participants were asked to perform assessments in each COVID-19 phase. For analyses, we included the average of all assessments per participant within each COVID-19 phase, providing a comprehensive representation of their monitoring data during that period.

**Table 1:** Average Monitoring Periods per Subject in Each COVID-19 Phase.

COVID-19 phase	Mean (std)	Persons
Pre-COVID-19	10.92 (8.06)	95
COVID-19 lockdown	2.26 (1.01)	47
COVID-19 de-escalation	2.54 (1.00)	46
COVID-19 relaxation	4.62 (2.62)	50

### 2.4 Data analysis

Descriptive analyses used frequencies and unweighted percentages for categorical variables, and means and standard deviations for continuous variables.

Linear mixed-model with random effects of participants and estimated with maximum likelihood (ML) method, using library nlme in R were conducted to evaluate the association of sedentary behavior and each physical activity intensity (light and MVPA) with depressive symptoms severity (Pinheiro and Bates, 2000; R Core Team, 2023). We considered the pandemic restrictions and the lockdown as a categorical variable in the model (hereafter COVID-19 phases).

We analyzed the longitudinal relationship of sedentary behavior, physical activity (light physical activity, and MVPA) with depressive symptoms (PHQ-8 scores); results presented in the Table 2 and in section 3.1. All analysis presented in Table 2 included all 1,491 observations from 95 participants who were monitored longitudinally between November 1st, 2019 and October 16th, 2020. As described previously, each participant could have been monitored in multiple opportunities in each COVID-19 phase, but only one observation per phase for each participant was included in the model. In the model 1 of the Table 2, each of the exposures (sedentary behavior, light physical activity, and MVPA) were evaluated in separate models in relation to depressive symptoms. In the model 2 of the Table 2, sedentary behavior and light PA models were adjusted by MVPA, whereas the MVPA model was adjusted by sedentary behavior. Analysis in the models 1 and 2 in the Table 2 were also adjusted by sex, age, comorbidity and monitoring phase. There were no significant differences in sociodemographic variables between participants across different COVID-19 phases.

**Table 2:** Longitudinal associations of sedentary behavior, light physical activity (light PA) and moderate-to-vigorous PA (MVPA) with depressive symptoms (PHQ-8 score) in participants with MDD in Spain.

Model 1					Model 2				
PHQ-8	*ß* (score of PHQ-8)	*β*std	(95% CI)	*p*-value	PHQ-8	*ß* (score of PHQ-8)	βstd	(95% CI)	*p*-value
Sedentary behavior	0.29	0.055	(0.10 to 0.47)	0.002	Sedentary behavior	0.37	0.071	(0.15 to 0.60)	0.001
Light PA	−0.37	−0.059	(−0.59 to −0.15)	0.001	Light PA	−0.35	−0.059	(−0.57 to −0.12)	0.003
MVPA	−0.003	−0.013	(−0.01 to 0.005)	0.430	MVPA	0.01	0.026	(−0.004 to 0.017)	0.211

PA refers to physical activity; Model 1: each of the exposures were evaluated in separate models; Model 2: Sedentary behavior and Light PA models were further adjusted by MVPA, whereas the MVPA model was adjusted by sedentary behavior. Models 1 and 2 were adjusted by sex, age, comorbidity and monitoring phase; CI refers to confidence interval.

**Figure 1:**
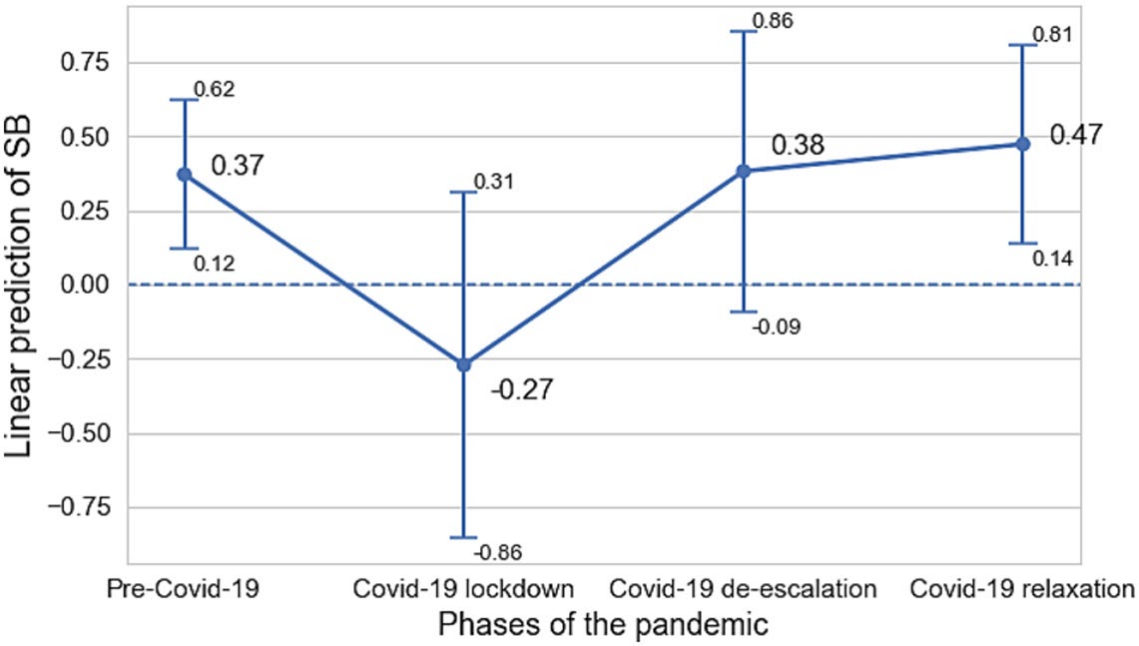
Longitudinal association between sedentary behavior and depressive severity across COVID-19 phases. Values are represented as average marginal effects and 95% confidence intervals.

**Figure 2:**
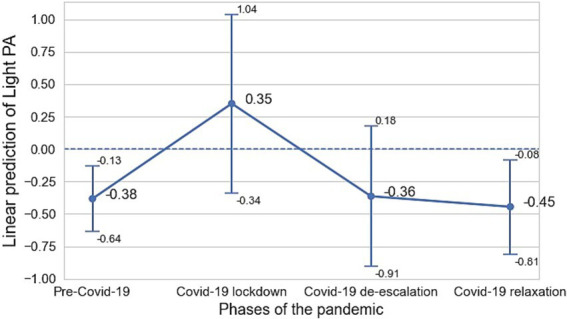
Longitudinal association between light PA and depressive severity across COVID-19 phases. Values are represented as average marginal effects and 95% confidence intervals.

In addition, we ran an linear mixed-model with random effects of participants and adjusted by ML method to evaluate whether the association of sedentary behavior and physical activity intensities (light and MVPA) with depressive symptoms differed depending on the COVID-19 phase; analyses described in the subsection 3.2. Figure 1 presents the association of sedentary behavior and MVPA with depressive symptoms across different COVID-19 phases whereas Figure 2 for light physical activity and MVPA with depressive symptoms across different COVID-19 phases. Both models included an interaction term between the exposures and the COVID-19 phase to estimate the strength of the association within each COVID-19 phase while accounting for the longitudinal structure of the data. All linear mixed-model analyses were adjusted by age, sex and comorbidity of other medical illness (Akhtar-Danesh and Landeen, 2007; Aluoja et al., 2004; Gutiérrez-Rojas et al., 2020). In the first step, we attempted to adjust for education level and marital status, but these variables did not contribute significantly to the model. We applied an ANOVA analysis to confirm that there were no significant differences when these variables were included or excluded.

In all multivariable models, we accepted a type I error of 5%.

## 3 Results

In total, 95 adults were included in the present study with; mean age of 53.0 (±10.2) years, 69.5% were female, 60.0% were cohabiting or married and 57.9% had comorbidities at baseline. The majority (73.7%) exhibited depression before the COVID-19 pandemic and were regular on antidepressant medications, with a mean PHQ-8 score of 13.7 (±5.98) points (Table 3). The number of participants monitored was 47 in the COVID-19 lockdown, 46 in the COVID-19 de-escalation and 50 in the COVID-19 relaxation phase. We did not observe attrition bias at follow up compared to baseline characteristics.

**Table 3:** Sociodemographic characteristics at baseline.

Characteristic	Pre-COVID-19 (*n* = 95)
Age, in years Mean (SD)	53.0 (±10.2)
Sex *n* (%)	
Female	66 (69.5%)
Male	29 (30.5%)
Marital status *n* (%)	
With a partner	57 (60.0%)
Without a partner (Single/Separated, Divorced/Widowed)	38 (40.0%)
Comorbidity with other medical illness *n* (%)	
Yes	55 (57.9%)
No	40 (42.1%)
Depression *n* (%)	
Yes	70 (73.7%)
No	25 (26.3%)

SD refers to standard deviation, Depression as measured by PHQ-8.

Table 4 presents the average of depression score, time in sedentary behavior and in distinct physical activity intensities in each of the monitoring periods.

**Table 4:** Depressive symptoms (PHQ-8 score), and time spent in sedentary behavior and in different physical activity intensities across COVID-19 phases.

	Pre-COVID-19	COVID-19 lockdown	COVID-19 de-escalation	COVID-19 relaxation
Depressive symptoms, PHQ-8 score	13.7 (5.98)	14.0 (6.80)	13.9 (6.71)	12.6 (6.78)
Sedentary behavior, hours/day	13.2 (1.08)	14.0 (0.95)	13.7 (1.17)	13.3 (1.19)
Light PA, hours/day	2.42 (0.90)	1.77 (0.80)	1.92 (1.02)	2.32 (1.07)
Moderate PA, min/day	13.3 (13.0)	8.27 (12.5)	9.56 (11.9)	11.8 (11.5)
Vigorous PA, min/day	10.3 (9.69)	5.44 (8.81)	10.7 (14.3)	10.7 (11.0)
Moderate-to-vigorous PA, min/day	23.6 (19.8)	13.7 (19.0)	20.3 (23.1)	22.5 (18.8)

Data presented as means and (standard deviations). PHQ-8 score ranges between 0 and 24 points. PA refers to physical activity.

### 3.1 Longitudinal association of sedentary behavior and physical activity (light and MVPA) with depressive symptoms in participants with MDD in Spain

An additional hour/day of sedentary behavior was longitudinally associated with higher depressive symptoms (*ß* = 0.29 score of PHQ-8, 95% CI 0.10 to 0.47; Table 2, model 1), whereas an additional hour/day in light physical activity was associated with lower depressive symptoms (*ß* = −0.37 score of PHQ-8, 95% CI −0.59 to −0.15; Table 2, model 1). Time in MVPA was not associated with depressive symptoms. Both sedentary behavior and light physical activity continued to be associated with depressive severity independent of time spent in MVPA (Table 2, model 2). Supplementary Table S1 presents the coefficients from Model 1 of Table 2, including the coefficients for the confounders that adjusted the models.

### 3.2 Longitudinal association of sedentary behavior and physical activity (light and MVPA) with depressive symptoms across monitoring phases

The association of sedentary behavior and light physical activity with depressive symptoms varied depending on the monitoring period, whereas MVPA was not associated with depressive symptoms in any of the monitoring phases. Particularly, both sedentary behavior (Figure 1) and light physical activity (Figure 2) were associated with depressive symptoms at the pre-COVID-19 phase and during the COVID-19 relaxation periods. Sedentary behavior was associated with increased depression in the pre-COVID-19 phase (*β* = 0.37, CI 0.12–0.62) and during the COVID-19 relaxation phase (*β* = 0.47 CI 0.14–0.81). On the other hand, light physical activity was associated with decreased depression in the pre-COVID-19 phase (*β* = −0.38, CI –0.64 – –0.13) and during the COVID-19 relaxation phase (*β* = −0.45, CI –0.81 – –0.08) (Table 5).

**Table 5:** The STROBE checklist of the study.

	Item No.	Recommendation	Page No.
Title and abstract	1	(a) Indicate the study’s design with a commonly used term in the title or the abstract	1
		(b) Provide in the abstract an informative and balanced summary of what was done and what was found	2
Introduction			
Background/rationale	2	Explain the scientific background and rationale for the investigation being reported	3
Objectives	3	State specific objectives, including any prespecified hypotheses	3–4
Methods			
Study design	4	Present key elements of study design early in the paper	4
Setting	5	Describe the setting, locations, and relevant dates, including periods of recruitment, exposure, follow-up, and data collection	4–6
Participants	6	(a) *Cohort study*—Give the eligibility criteria, and the sources and methods of selection of participants. Describe methods of follow-up *Case–control study*—Give the eligibility criteria, and the sources and methods of case ascertainment and control selection. Give the rationale for the choice of cases and controls *Cross-sectional study*—Give the eligibility criteria, and the sources and methods of selection of participants	4–6
		(b) *Cohort study*—For matched studies, give matching criteria and number of exposed and unexposed *Case–control study*—For matched studies, give matching criteria and the number of controls per case	
Variables	7	Clearly define all outcomes, exposures, predictors, potential confounders, and effect modifiers. Give diagnostic criteria, if applicable	6
Data sources/measurement	8	For each variable of interest, give sources of data and details of methods of assessment (measurement). Describe comparability of assessment methods if there is more than one group	*6*
Bias	9	Describe any efforts to address potential sources of bias	6–7
Study size	10	Explain how the study size was arrived at	5–6

## 4 Discussion

In this study, we investigated the longitudinal association of sedentary behavior as well as different physical activity intensities with depressive symptoms in a cohort of Spanish adults with a recent history of MDD across COVID-19 phases. Sedentary behavior, positively, and light physical activity, negatively, were associated with depressive symptoms in participants with a history of MDD, whereas time in MVPA was not (results presented in Table 2). Furthermore, we observed that an additional hour/day of sedentary behavior is related to higher depressive severity during the pre-COVID-19 and the relaxation phases (results presented in Figure 1). Besides, an extra hour/day of light physical activity is associated with lower depressive symptomatology during the same phases (results presented in Figure 2), whereas MVPA was not associated with depressive symptoms in any COVID-19 phases.

Although sedentary behavior and light physical activity were determinants of depressive symptoms, their importance in relation to depressive symptomatology in adults with MDD was weakened during the strictest periods of the COVID-19 pandemic. Of note, both sedentary behavior and light physical activity returned to be associated with depressive symptoms after easing the COVID-19 restrictions. It is possible that other factors related to the pandemic (worry of being infected, social isolation, unemployment, economic losses, changes in daily routines and social dynamics) were more relevant to their mental health during the most restrictive phases of the pandemic. It is also possible that the restrictions due to the COVID-19 pandemic restricted people from performing physical activities.

Our findings suggest that light physical activity might be more relevant than MVPA in relation to the depressive symptomatology of adults with a history of MDD. Of note, a recent systematic review and network meta-analysis of randomized controlled trials concluded that exercises prescribed in higher intensities showed higher reduction in depressive symptomatology in adults with clinical cut-offs for major depression (Noetel et al., 2024). To date, the state-of-the-art did not reach a consensus on the varying impact of different intensities of physical activity on depressive symptoms.

Experimental studies demonstrate that exercise, whether of lower or higher intensity, positively affects depressive symptoms in adults with depression (Hanssen et al., 2018; Noetel et al., 2024) and those with major depressive disorder (MDD) (Minghetti et al., 2018; Nasstasia et al., 2019; Schuch and Stubbs, 2019; Stubbs et al., 2018). Some studies suggest a more significant impact on depressive symptoms with higher exercise intensities (Hanssen et al., 2018; Noetel et al., 2024). It is speculated that higher intensities may lead to greater reductions in depressive symptoms due to enhanced physiological responses, such as reduced inflammation, oxidative stress, and increased neuronal regeneration (Schuch and Stubbs, 2019; Stubbs et al., 2018; Kandola et al., 2019).

Observational studies using questionnaires to assess physical activity levels report stronger associations between moderate-to-vigorous physical activity (MVPA) and reduced depressive symptoms compared to light physical activity (LPA) (Dishman et al., 2021; Wolf et al., 2021; Schuch et al., 2018). However, a recent study using accelerometry found that LPA was more protective against incident depression than higher intensities (Lima et al., 2024). This divergence may be attributed to the methods of physical activity assessment. Previous research has shown conflicting health associations depending on whether physical activity was assessed via questionnaires or accelerometers (Gill et al., 2023).

Questionnaires, relying on self-reported data, are prone to recall bias and social desirability bias, which can lead to inaccurate estimations of activity levels. Adults with lower physical activity levels might perceive and report low-intensity activities as higher intensity due to their lower fitness levels and subjective experiences, further distorting the data. In contrast, accelerometers provide objective, continuous data, reducing reporting bias and offering a more accurate measure of overall physical activity. However, they often fail to capture specific activity types, such as swimming or cycling, and may miss context-specific nuances of physical activity patterns. These discrepancies in data capture methods can significantly influence the observed associations between physical activity and health outcomes in studies (Gill et al., 2023).

Psychosocial mechanisms might explain the association between LPA and depressive symptoms in adults with MDD (Kandola et al., 2019; Schuch et al., 2018). In particular, a person with MDD might be more likely to perform a physical activity in the company of a partner, a friend or a family member in lower physical activity intensity, strengthening their social support and self-esteem, which might decrease their depressive symptomatology (Kandola et al., 2019; Schuch et al., 2018). Noteworthy, the lack of association between MVPA and depressive symptoms in the current study might be due to the limited number of minutes in MVPA, which could have been too discreet to provide mental health benefits. Nevertheless, participation in MVPA should also be encouraged because of the numerous health benefits and our results do not suggest that participation in MVPA is deleterious to the mental health of patients with MDD (Hallgren et al., 2016; Schuch et al., 2018; Sheikh et al., 2018).

Notwithstanding the strengths of our study, it does have some limitations. First, participant adherence to the study protocol (i.e., questionnaire completion or wearing de wristband device) decreases during the most restrictive periods which contributed to the limited number of participants included in the analyses (*n* = 95). Nevertheless, our analysis included all the information available on each participant who contributed with data in at least one monitoring period. Importantly, the use of direct assessment of physical activity during different monitoring periods, including the pandemic, overcome a pivotal limitation of previous studies.

Second, participants recruited at different times may use different devices for smartphones and Fitbit depending on the availability and enrolment dates, which might have impacted on the estimation of physical activity and sedentary behavior. Nevertheless, recent meta-analyses observed that Fitbit model is not a significant factor impacting on the device validity (Leung et al., 2022).

Third, while our longitudinal study provides valuable insights into the relationship between physical activity levels and depressive symptoms among adults with major depressive disorders, the potential for reverse causality, selection bias and observation bias should be acknowledged as they may influence the generalizability and accuracy of our findings.

## 5 Conclusion

Sedentary behavior, positively, and light physical activity, negatively, were associated with depressive symptoms in participants with a history of MDD, whereas time in MVPA was not. The incorporation of light physical activity should be stimulated by mental health professionals, future public health programs and research interventions aiming to decrease depressive symptomatology in adults with a history of MDD, especially by diminishing time in sedentary activities.

## Data Availability

The raw data supporting the conclusions of this article will be made available by the authors on reasonable request, without undue reservation.

## Footnotes

^1^ https://www.kcl.ac.uk/research/radarcns

^2^ http://radar-base.org/

## RADAR CNS consortium

https://www.kcl.ac.uk/research/radarcns

## Group members of RADAR-MDD-Spain

Peñarrubia-Maria Maria Teresa, Consortium for Biomedical Research in Epidemiology and Public Health (CIBERESP), Instituto de Salud Carlos III, Madrid, Spain, Unitat de Suport a la Recerca Regió Metropolitana Sud, Fundació Institut Universitari per a la Recerca a l’Atenció Primària de Salut Jordi Gol i Gurina (IDIAPJGol), Barcelona, Spain; Gardeñes Moron Lluisa, Unitat de Suport a la Recerca Regió Metropolitana Sud, Fundació Institut Universitari per a la Recerca a l’Atenció Primària de Salut Jordi Gol i Gurina (IDIAPJGol), Barcelona, Spain; Jugo Beatriz, Unitat de Suport a la Recerca Regió Metropolitana Sud, Fundació Institut Universitari per a la Recerca a l’Atenció Primària de Salut Jordi Gol i Gurina (IDIAPJGol), Barcelona, Spain; Orive Cristina, Unitat de Suport a la Recerca Regió Metropolitana Sud, Fundació Institut Universitari per a la Recerca a l’Atenció Primària de Salut Jordi Gol i Gurina (IDIAPJGol), Barcelona, Spain; De Miguel Blanca, Unitat de Suport a la Recerca Regió Metropolitana Sud, Fundació Institut Universitari per a la Recerca a l’Atenció Primària de Salut Jordi Gol i Gurina (IDIAPJGol), Barcelona, Spain; Blanco Elena, Unitat de Suport a la Recerca Regió Metropolitana Sud, Fundació Institut Universitari per a la Recerca a l’Atenció Primària de Salut Jordi Gol i Gurina (IDIAPJGol), Barcelona, Spain; Gallardo Cristina, Unitat de Suport a la Recerca Regió Metropolitana Sud, Fundació Institut Universitari per a la Recerca a l’Atenció Primària de Salut Jordi Gol i Gurina (IDIAPJGol), Barcelona, Spain; Cobo-Guerrero Silvia, Unitat de Suport a la Recerca Regió Metropolitana Sud, Fundació Institut Universitari per a la Recerca a l’Atenció Primària de Salut Jordi Gol i Gurina (IDIAPJGol), Barcelona, Spain; Molero Aida, Unitat de Suport a la Recerca Regió Metropolitana Sud, Fundació Institut Universitari per a la Recerca a l’Atenció Primària de Salut Jordi Gol i Gurina (IDIAPJGol), Barcelona, Spain; Belvis Mariscal Cristina, Unitat de Suport a la Recerca Regió Metropolitana Sud, Fundació Institut Universitari per a la Recerca a l’Atenció Primària de Salut Jordi Gol i Gurina (IDIAPJGol), Barcelona, Spain; Martinez Gemma, Unitat de Suport a la Recerca Regió Metropolitana Sud, Fundació Institut Universitari per a la Recerca a l’Atenció Primària de Salut Jordi Gol i Gurina (IDIAPJGol), Barcelona, Spain; Valdés Raúl, Unitat de Suport a la Recerca Regió Metropolitana Sud, Fundació Institut Universitari per a la Recerca a l’Atenció Primària de Salut Jordi Gol i Gurina (IDIAPJGol), Barcelona, Spain; Torres-Martinez Rebecca, Unitat de Suport a la Recerca Regió Metropolitana Sud, Fundació Institut Universitari per a la Recerca a l’Atenció Primària de Salut Jordi Gol i Gurina (IDIAPJGol), Barcelona, Spain; López Fran, Unitat de Suport a la Recerca Regió Metropolitana Sud, Fundació Institut Universitari per a la Recerca a l’Atenció Primària de Salut Jordi Gol i Gurina (IDIAPJGol), Barcelona, Spain; Mera-Cordero Francisco, Unitat de Suport a la Recerca Regió Metropolitana Sud, Fundació Institut Universitari per a la Recerca a l’Atenció Primària de Salut Jordi Gol i Gurina (IDIAPJGol), Barcelona, Spain; Soraluce Acebo Ramón, Unitat de Suport a la Recerca Regió Metropolitana Sud, Fundació Institut Universitari per a la Recerca a l’Atenció Primària de Salut Jordi Gol i Gurina (IDIAPJGol), Barcelona, Spain; Sarró Marta, Unitat de Suport a la Recerca Regió Metropolitana Sud, Fundació Institut Universitari per a la Recerca a l’Atenció Primària de Salut Jordi Gol i Gurina (IDIAPJGol), Barcelona, Spain; Moliner-Molina Cristina, Unitat de Suport a la Recerca Regió Metropolitana Sud, Fundació Institut Universitari per a la Recerca a l’Atenció Primària de Salut Jordi Gol i Gurina (IDIAPJGol), Barcelona, Spain; Flores Maria Rosa, Unitat de Suport a la Recerca Regió Metropolitana Sud, Fundació Institut Universitari per a la Recerca a l’Atenció Primària de Salut Jordi Gol i Gurina (IDIAPJGol), Barcelona, Spain; Sostres Susana, Unitat de Suport a la Recerca Regió Metropolitana Sud, Fundació Institut Universitari per a la Recerca a l’Atenció Primària de Salut Jordi Gol i Gurina (IDIAPJGol), Barcelona, Spain; Ait Ben Aissa, Unitat de Suport a la Recerca Regió Metropolitana Sud, Fundació Institut Universitari per a la Recerca a l’Atenció Primària de Salut Jordi Gol i Gurina (IDIAPJGol), Barcelona, Spain; Poch Marta, Unitat de Suport a la Recerca Regió Metropolitana Sud, Fundació Institut Universitari per a la Recerca a l’Atenció Primària de Salut Jordi Gol i Gurina (IDIAPJGol), Barcelona, Spain; Olmos Maria Cristina, Unitat de Suport a la Recerca Regió Metropolitana Sud, Fundació Institut Universitari per a la Recerca a l’Atenció Primària de Salut Jordi Gol i Gurina (IDIAPJGol), Barcelona, Spain; Ortiz Navarrete Sónia, Unitat de Suport a la Recerca Regió Metropolitana Sud, Fundació Institut Universitari per a la Recerca a l’Atenció Primària de Salut Jordi Gol i Gurina (IDIAPJGol), Barcelona, Spain; Martinez Andrea, Unitat de Suport a la Recerca Regió Metropolitana Sud, Fundació Institut Universitari per a la Recerca a l’Atenció Primària de Salut Jordi Gol i Gurina (IDIAPJGol), Barcelona, Spain; Freixas Olga, Unitat de Suport a la Recerca Regió Metropolitana Sud, Fundació Institut Universitari per a la Recerca a l’Atenció Primària de Salut Jordi Gol i Gurina (IDIAPJGol), Barcelona, Spain; Caldón Beatriz, Unitat de Suport a la Recerca Regió Metropolitana Sud, Fundació Institut Universitari per a la Recerca a l’Atenció Primària de Salut Jordi Gol i Gurina (IDIAPJGol), Barcelona, Spain; Cano Maria Isabel, Unitat de Suport a la Recerca Regió Metropolitana Sud, Fundació Institut Universitari per a la Recerca a l’Atenció Primària de Salut Jordi Gol i Gurina (IDIAPJGol), Barcelona, Spain; Lombardini Federica, Research, Innovation and Teaching Unit, Parc Sanitari Sant Joan de Déu, Sant Boi de Llobregat, Spain; Coromina Marta, Research, Innovation and Teaching Unit, Parc Sanitari Sant Joan de Déu, Sant Boi de Llobregat, Spain; Arranz Belen, Research, Innovation and Teaching Unit, Parc Sanitari Sant Joan de Déu, Sant Boi de Llobregat, Spain; Arranz Sara, Research, Innovation and Teaching Unit, Parc Sanitari Sant Joan de Déu, Sant Boi de Llobregat, Spain; Salvador Laia, Research, Innovation and Teaching Unit, Parc Sanitari Sant Joan de Déu, Sant Boi de Llobregat, Spain; Hernandez Carla, Research, Innovation and Teaching Unit, Parc Sanitari Sant Joan de Déu, Sant Boi de Llobregat, Spain; Marqueta-Marqués Cristina, Research, Innovation and Teaching Unit, Parc Sanitari Sant Joan de Déu, Sant Boi de Llobregat, Spain; Calamardo Elisabet, Research, Innovation and Teaching Unit, Parc Sanitari Sant Joan de Déu, Sant Boi de Llobregat, Spain; Ferenc Tamas, Research, Innovation and Teaching Unit, Parc Sanitari Sant Joan de Déu, Sant Boi de Llobregat, Spain; Rubio-Alacid Elena, Research, Innovation and Teaching Unit, Parc Sanitari Sant Joan de Déu, Sant Boi de Llobregat, Spain; Rojas Antonio, Research, Innovation and Teaching Unit, Parc Sanitari Sant Joan de Déu, Sant Boi de Llobregat, Spain; Druetta Mauro, Research, Innovation and Teaching Unit, Parc Sanitari Sant Joan de Déu, Sant Boi de Llobregat, Spain; Marso Esperança, Research, Innovation and Teaching Unit, Parc Sanitari Sant Joan de Déu, Sant Boi de Llobregat, Spain; Hernandez-Fernandez Elisabet, Research, Innovation and Teaching Unit, Parc Sanitari Sant Joan de Déu, Sant Boi de Llobregat, Spain; Cuñat Oriol, Research, Innovation and Teaching Unit, Parc Sanitari Sant Joan de Déu, Sant Boi de Llobregat, Spain; Otero Begoña, Research, Innovation and Teaching Unit, Parc Sanitari Sant Joan de Déu, Sant Boi de Llobregat, Spain; Tronchoni Mónica, Research, Innovation and Teaching Unit, Parc Sanitari Sant Joan de Déu, Sant Boi de Llobregat, Spain; Tessari Diego, Research, Innovation and Teaching Unit, Parc Sanitari Sant Joan de Déu, Sant Boi de Llobregat, Spain; Labarta Eugenia, Research, Innovation and Teaching Unit, Parc Sanitari Sant Joan de Déu, Sant Boi de Llobregat, Spain; Delisau Yaiza, Research, Innovation and Teaching Unit, Parc Sanitari Sant Joan de Déu, Sant Boi de Llobregat, Spain; Angelats Marina, Research, Innovation and Teaching Unit, Parc Sanitari Sant Joan de Déu, Sant Boi de Llobregat, Spain; Vilella Elisabet, FEA Psicología Clínica, HU Institut Pere Mata, Vendrell, Spain; Llaosa-Scholten Raúl, FEA Psicología Clínica, HU Institut Pere Mata, Vendrell, Spain; Adam Noemí, FEA Psicología Clínica, HU Institut Pere Mata, Vendrell, Spain, Reneses Prieto Blanca, Servicio de Psiquiatría. Hospital Clínico San Carlos, Madrid, Spain; and Zamarro Arranz Maria Luisa, Servicio de Psiquiatría. Hospital Clínico San Carlos, Madrid, Spain.
