# Supplementary material for: Pre- and post-COVID 19 outbreak relationship between physical activity and depressive symptoms in Spanish adults with major depressive disorder: a secondary analysis of the RADAR-MDD cohort study
Source: Front Psychol. 2024 Nov 12;15:1436611. doi: 10.3389/fpsyg.2024.1436611 (PMC11599828; doi:10.3389/fpsyg.2024.1436611)
Supplement: Supplementary file 1 [file Table_1.DOCX]

**Supplementary Table 1. Longitudinal associations of sedentary behaviour, light physical activity (light PA) and moderate-to-vigorous PA (MVPA) with depressive symptoms (PHQ-8 score) in participants with MDD in Spain.**

Supplementary Table 1 presents the coefficients from Model 1 of Table 4, including the coefficients for the confounders that adjusted the models.

| **Model 1 – Sedentary behaviour model** | | | | | | | |
| --- | --- | --- | --- | --- | --- | --- | --- |
| **PHQ-8** | **ß** | | **(95% CI)** | | | **p-value** | |
| **Sedentary behaviour** | **0.29** | | **0.10-0.47** | | | **0.002** | |
| **Sex (female)** | **2.96** | | **0.53 – 5.40** | | | **0.017** | |
| **Age** | **-0.07** | | **-0.18- 0.04** | | | **0.240** | |
| **Comorbility** | 3.66 | | 1.38 – 5.94 | | | 0.002 | |
| **Covid-19 lockdown** | 0.88 | | 0.29-1.47 | | | 0.003 | |
| **Covid-19 de-escalation** | 0.67 | | 0.11-1.22 | | | 0.020 | |
| **Covid-19 relaxation phase** | 0.06 | | -0.037- 0.049 | | | 0.778 | |
| **Model 1 – LPA model** | | | | | | |  |
| **PHQ-8** | | **ß** | | **(95% CI)** | **p-value** | |  |
| **Light PA** | | **-0.37** | | **-0.59 - -0.15** | **0.001** | |  |
| **Sex (female)** | | **3.05** | | **0.62 – 5.49** | **0.014** | |  |
| **Age** | | **-0.07** | | **-0.18 – 0.04** | **0.240** | |  |
| **Comorbility** | | 3.65 | | 1.37 – 5.93 | 0.002 | |  |
| **Covid-19 lockdown** | | 0.88 | | 0.29 – 1.47 | 0.003 | |  |
| **Covid-19 de-escalation** | | 0.65 | | 0.09- 1.21 | 0.024 | |  |
| **Covid-19 relaxation phase** | | 0.06 | | -0.37 – 0.49 | 0.777 | |  |
| **Model 1 – MVPA model** | | | | | | |  |
| **PHQ-8** | | **ß** | | **(95% CI)** | **p-value** | |  |
| **MVPA** | | **-0.003** | | **-0.01- 0.005** | **0.430** | |  |
| **Sex (female)** | | **2.87** | | **0.41 – 5.43** | **0.022** | |  |
| **Age** | | **-0.07** | | **-0.18 – 0.04** | **0.235** | |  |
| **Comorbility** | | 3.67 | | 1.37 – 5.98 | 0.002 | |  |
| **Covid-19 lockdown** | | 1.07 | | 0.49 – 1.65 | <0.001 | |  |
| **Covid-19 de-escalation** | | 0.83 | | 0.27 – 1.38 | 0.003 | |  |
| **Covid-19 relaxation phase** | | 0.13 | | -0.30- 0.56 | 0.564 | |  |
